# New Insights into Pathophysiology and New Risk Factors for ACS

**DOI:** 10.3390/jcm12082883

**Published:** 2023-04-14

**Authors:** Matteo Nardin, Monica Verdoia, Nicola Laera, Davide Cao, Giuseppe De Luca

**Affiliations:** 1 Department of Biomedical Sciences, Humanitas University, 20072 Milan, Italy; 2 Third Medicine Division, Department of Medicine, ASST Spedali Civili, 25123 Brescia, Italy; 3 Division of Cardiology, Ospedale degli Infermi, ASL Biella, 13900 Biella, Italy; 4 Department of Translational Medicine, Eastern Piedmont University, 13100 Novara, Italy; 5 Department of Clinical and Experimental Sciences, University of Brescia, 25121 Brescia, Italy; 6 Division of Cardiology, AOU “Policlinico G. Martino”, Department of Clinical and Experimental Medicine, University of Messina, 98166 Messina, Italy; 7 Division of Cardiology, IRCCS Hospital Galeazzi—Sant’Ambrogio, 20161 Milan, Italy

**Keywords:** acute coronary syndrome, pathophysiology, risk factor, genetics, inflammation

## Abstract

Cardiovascular disease still represents the main cause of mortality worldwide. Despite huge improvements, atherosclerosis persists as the principal pathological condition, both in stable and acute presentation. Specifically, acute coronary syndromes have received substantial research and clinical attention in recent years, contributing to improve overall patients’ outcome. The identification of different evolution patterns of the atherosclerotic plaque and coronary artery disease has suggested the potential need of different treatment approaches, according to the mechanisms and molecular elements involved. In addition to traditional risk factors, the finer portrayal of other metabolic and lipid-related mediators has led to higher and deep knowledge of atherosclerosis, providing potential new targets for clinical management of the patients. Finally, the impressive advances in genetics and non-coding RNAs have opened a wide field of research both on pathophysiology and the therapeutic side that are extensively under investigation.

## 1. Introduction

A number of substantial improvements has been achieved in the diagnosis and treatment of acute coronary syndrome (ACS) in the last decades, especially in terms of antithrombotic therapies [1,2] and percutaneous intervention [3,4,5,6]. However, cardiovascular diseases persist as the leading cause of mortality worldwide, with ischemic heart disease accounting for the majority of cardiovascular deaths [7,8].

Angiographically identifiable atherosclerotic lesions are considered the key elements in the determinism of coronary artery disease (CAD): historically, clinically significant (flow-limiting) coronary stenoses were the primary lesions for chronic coronary syndrome (CCS), with their future progression causing acute cardiac events, including unstable angina, myocardial infarction (MI) and cardiac death.

Although the revascularization of severe coronary artery stenoses certainly reduces symptoms and ameliorates quality of life, several reports have found no substantial prognostic improvements [9,10] of interventional approaches, while optimal medical therapy has demonstrated a reduction in cardiac death and MI [9,11]. Despite guideline-based treatment, a proportion of patients with CCS progress to acute event with detrimental consequences in overall patient outcome.

Thus, research efforts have shifted to the identification of features and characteristics of atherosclerotic plaques, aiming to better patient risk stratification [12,13,14,15]. Alongside that, advances in understanding pathogenic pathways and genetic role have provided new insights into the atherothrombotic process.

## 2. Definition and Clinical Presentation of Acute Coronary Syndrome

The clinical spectrum of ACS includes unstable angina, non-ST-segment elevation myocardial infarction (NSTEMI) and ST-segment elevation myocardial infarction (STEMI). These three clinical entities are graduated according to the severity and rapidity of the required action for their treatment. At the bottom of severity is placed unstable angina, in which the referred symptoms suggest an acute myocardial injury, but the biochemical evidence is lacking. NSTEMI and STEMI are grouped in the nosological condition of type 1 MI, according to the Fourth Universal Definition of Myocardial Infarction, requiring appreciable troponin level changes that both rise or fall together with clinical evidence of ischemia [16].

**Figure 1:**
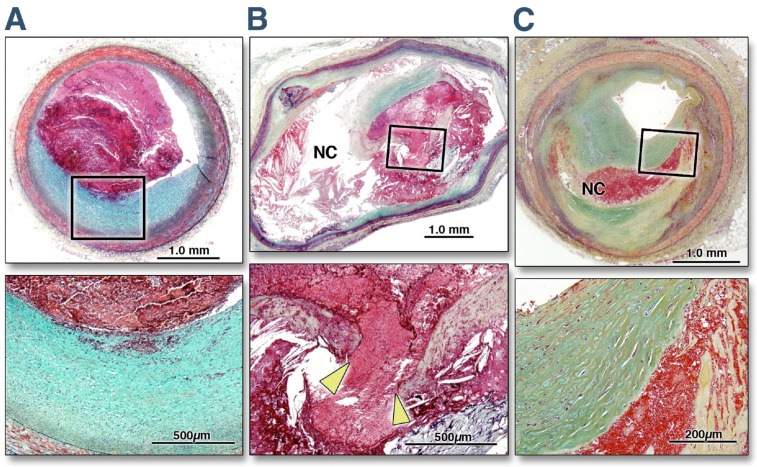
Histomorphological Characteristics of plaque erosion, plaque rupture, and a stable plaque. The top three photos report the cross-sectional area of a culprit lesion displaying a plaque erosion in a patient with acute coronary syndrome (**A**), a culprit lesion displaying a plaque disruption in a patient with acute coronary syndrome (**B**), and a stable unrelated plaque from a non-culprit site in another patient dying from sudden cardiac death (**C**). The boxed areas in the top panels are magnified in the bottom panels for better histological characterization. (**A**) The eroded plaque shows subcritical stenosis, an unremarkable necrotic core, and overlying thrombus on an intact fibrous cap. The cap is rich in smooth muscle cells and proteoglycans, and there is minimal inflammation at the base of the thrombus. The plaque does not show any positive remodeling. (**B**) Conversely, a positively remodeled, critically occlusive atherosclerotic plaque with a cholesterol crystal-rich large necrotic core (NC) covered by a very thin and inflamed fibrous cap, which is disrupted (area between the arrowheads). Smooth muscle cells are visible in the medial layer and thin fibrous cap and are minimally present at the base of the neointima. A large thrombogenic necrotic core is in communication with the vessel lumen with an occlusive thrombus. (**C**) A stable plaque shows smooth muscle and collagen-rich histology. The hemorrhagic necrotic core in the middle that separates the collagen of two separate ages represents a healed rupture site. The lesion is critically narrowed but does not show any positive remodeling or overlying thrombus. Reprinted from [25], with permission from Elsevier.

Even if the proportion of STEMI among ACS is decreasing in Western countries, thanks to improvements in contrasting well-established risk factors, the rates of in-hospital mortality and morbidity remain high [17]. Moreover, the previous paradigm of coronary atherosclerotic plaque rupture as the singular cause of STEMI or NSTEMI, recently re-named non-ST-segment elevation acute coronary syndrome (NSTE-ACS), has fallen in the last decade [18]. The availability of intravascular ultrasonography (IVUS) and optical coherence tomography (OCT) has allowed for the study of the in vivo characteristics and morphology of plaques and their composition [19,20,21,22,23]. The majority of ACS has been caused by the rupture of an atherosclerotic plaque with thrombus formation. Studies among patients referred for an ACS have documented the rupture of lipid-rich plaque in two thirds of cases [24]. However, a non-negligible proportion of patients experience ACS due to plaque erosion, calcific nodules, coronary spasm, and spontaneous coronary artery dissection (Figure 1).

## 3. Physiopathology of Acute Coronary Syndrome

### 3.1. Plaque Disruption

The principal and more frequent event that leads to an acute presentation of coronary atherosclerosis is constituted by the luminal rupture of a “vulnerable” plaque [25]. Its features include a large lipid core mixed with foam cells, macrophages. That atheroma is covered by a thin fibrotic cap including extracellular matrix components. The acute rupture of the protective cap releases prothrombotic substances and material from the plaque, activating the coagulative cascade, thrombus formation with consequent ischemia [26]. For decades, research has focused their efforts on understanding the pathophysiology of the rupture of atheroma’s thin cap. Several inflammatory mediators, including interferon-γ and interleukin (IL)-1, block the production of extracellular matrix elements [27] and stimulate macrophages and other cells to release proteases that degrade extracellular molecules [28]. The same triggers that deteriorate the atheroma’s thin cap stimulate the production of prothrombotic elements, fibrin and plasminogen activator inhibitor-1, enhancing clot formation [29]. The exposed core elements, including von Willebrand factor, collagen and tissue factor, activate the circulating platelets, which in turn potentiate the coagulative process, resulting in a rapid thrombus formation and acute onset of heart ischemia [30,31].

### 3.2. Plaque Erosion

A relatively new concept is represented by the superficial erosion of atherosclerotic plaque as the cause of ACS. Historical data suggest a prevalence of 20% of ACS patients characterized by plaque erosion in the culprit lesions, while more recent reports indicate about 40% of ACS patients displaying plaque erosion [32,33,34]. Improvements in technological instruments, particularly the OCT, have helped to identify this condition in vivo. In general, patients with plaque erosion are younger than subjects admitted for ACS due to plaque rupture. Data from a large OCT systematic study showed 53.8 vs. 65.1 years, respectively, for patients with plaque erosion compared to those with plaque rupture [35]. The distribution of traditional cardiovascular risk factors is unbalanced between these two conditions. Patients with plaque erosion usually display lower prevalence of diabetes mellitus and hypertension, lower levels of low-density lipoprotein cholesterol and C-reactive protein, and a higher concentration of hemoglobin [36,37]. In addition, the complexity and severity of CAD are lower in patients with plaque erosion than among those with plaque rupture [38].

Considering the substantial differences observed from the clinical side, it is essential to understand the molecular mechanisms to tailor patient management and to explore new potential targets of treatment.

The first crucial issue is represented by the local shear stress corresponding to the plaque. Human studies have shown that a thrombus occurs in the zones of high endothelial shear stress [39]. The fluid dynamic impact leads to the degradation of the basement membrane, endothelial cell desquamation and death [40]. A contribution by innate immunity has been suggested [41]. Inflammation and minimally oxidized low-density lipoprotein (LDL) promote the expression of matrix metalloproteinase (MMP)-14 and MMP-2, which degrade type IV collagen, the main component of the basement membrane [42,43]. Furthermore, the enhanced activation of toll-like receptor (TLR)-2 has been found to maintain the underlying inflammation [44] and to favor the recruitment of granulocytes through the increased expression of leukocyte adhesion molecules [45]. The recruited granulocytes, mostly neutrophils, are prone to form neutrophil extracellular traps (NETs) that have been linked to the pathophysiology of plaque erosion and thrombus formation. NETs are constituted by cytokines and tissue factors, and they can entrap circulating platelets, thus promoting atherosclerosis [46]. Finally, impaired endothelium homeostasis has been implied in endothelial to mesenchymal transition, which contributes to plaque erosion and loss of integrity, together with overexpression of MMP-2, transforming growth factor-β (TGF-β), and vascular growth factor (VEGF) [47,48].

### 3.3. Calcified Nodules

Among less common causes of ACS, there are calcified nodules. Their frequencies range from 4% to 7% as the cause of ACS [49]. These heavily calcified lesions have been found to be more frequent among the elderly [50] and in those with chronic kidney disease [51,52]. From a pathological point of view, they are defined by the presence of a fracture in the calcified sheet mixed with fibrin and a disrupted fibrous cap with an overlying thrombus [53]; a histology study has shown that the break in the calcium sheet into the lumen might be the trigger. Negative remodeling has been observed in calcified nodule lesions more frequently than in lesions with ruptures of erosion [24].

### 3.4. Spontaneous Coronary Artery Dissection

Spontaneous coronary artery dissection (SCAD) is a condition characterized by an intimal tear leading to the creation of a false lumen in the coronary wall in absence of clear or known mechanical causes, including traumatic events or wire manipulation. The subsequent compression of the vessel lumen leads to ischemia in the post-dissection myocardial area [18]. Epidemiological data show an overall low prevalence of this condition, less than 5%, even if a higher incidence has been displayed in specific subgroup of patients, including pregnant or postpartum women [54]. The optimal treatment of SCAD has still not been determined. Routine percutaneous coronary intervention performance is associated with a particularly elevated rate of technical failure, due to wiring the false lumen or extension of the dissection [55].

### 3.5. Coronary Spasm

A still poorly understood cause of ACS without plaque rupture is represented by coronary artery spasm. Some hypotheses involve reduced nitric oxide availability due to endothelial dysfunction, leading to increased smooth muscle cell reactivity [56]. A proportion of about 25% of patients undergoing coronary angiography has shown coronary spasm [57]. This should be expected, particularly if coronary angiography provides negligible findings for significant stenosis [58]. The occurrence is higher among older men and postmenopausal women [57], and racial differences have been detected even if without a definite explanation, to date [59]. A previous study has inquired about coronary spasms induced by acetylcholine among patients with stable angina. The authors found a worse prognosis at 28 months among patients with a high degree of coronary spasm [60]. The lack of a systematic approach to diagnose coronary spasm hampers the availability of robust treatment guidelines, even if some authors suggest a routine use of acetylcholine to unhide coronary spasm in ACS patients with coronary angiography negative for clear culprit lesions [61].

## 4. Recent Known Advances on Immunity

### 4.1. NETs and NLRP3

Several studies have claimed a central role of neutrophils in CAD progression, including the abrupt presentation of an ACS [62]. The activation of NETs above atherosclerotic plaque together with nucleotide-binding oligomerization domain-like receptor family pyrin domain containing 3 (NLRP3) are known and accepted as key pathogenetic steps [63].

Neutrophil action in promoting atherosclerosis involves monocyte entry into atherosclerotic lesions and macrophage activation, while the released myeloperoxidase accelerates LDL oxidation and then foam cell formation [64]. The formation of NET is a relatively recent element related to neutrophils that worsen atherosclerosis [65]. The network of extracellular fibers composed by the DNA of neutrophils is activated by numerous stimuli involving ROS, factor crystallizable and complement receptors, TLR-2 and -4 signaling [66], with a consequent impact on platelet aggregation and macrophage release of IL-1β [67].

The NLRP3 inflammasome is a key mediator of inflammatory diseases, including atherosclerosis and other vascular diseases. Recent evidence has suggested that IL-1β-mediated inflammation drives atherothrombotic events, indicating that NLRP3 inflammasome is a major contributor to atherosclerosis.

**Figure 2:**
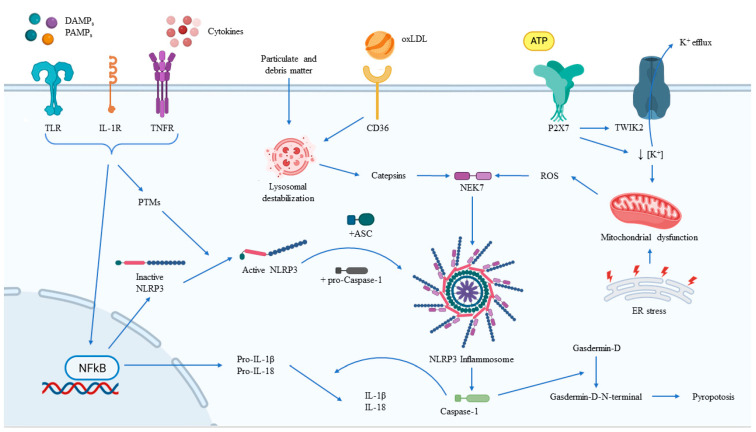
Regulation of NLRP3 inflammasome activation. The main external and intracellular factors involved in the activation and modulation of the NLRP3 inflammasome, together with its main effects. ASC, adaptor apoptosis-associated speck-like protein containing a caspase-recruitment domain; ATP, adenosine triphosphate; DAMPs, damage/danger-associated molecular patterns; ER, endoplasmic reticulum; IL-1R, interleukin-1 receptor; NFkB, nuclear factor-kB; NLRP3, nucleotide-binding oligomerization domain-like receptor family pyrin domain containing 3; oxLDL, oxide low-density lipoprotein; PAMPs, pathogen-associated molecular patterns; PTMs, post-translational modification; TLR, toll-like receptor; TNFR, tumor-necrosis factor receptor.

NLRP3 is known to be activated by a wide range of diverse stimuli, most of which have not been shown to directly interact with it (Figure 2). Thus, it is theorized that NLRP3 can sense cellular events that are triggered by activating stimuli. Common upstream events proposed for NLRP3 inflammasome activation include K^+^ efflux (decrease in intracellular K^+^), the generation of ROS derived from mitochondria, and cathepsin release from the lysosome. Mitochondria-derived ROS production has been demonstrated to be important for NLRP3 inflammasome activation, with studies suggesting that oxidized mitochondrial DNA released in response to NLRP3 activators can drive the activation. The role of NADPH oxidase in NLRP3 inflammasome activation is controversial, although it has been suggested that NADPH oxidase-4 may play a role in certain conditions. Furthermore, the release of cathepsin from damaged lysosomes is another cellular event that can trigger NLRP3 inflammasome activation. This is commonly caused by the presence of particulate matter such as monosodium urate and cholesterol crystals. These particles are taken up by macrophages, but their digestion in lysosomes is inadequate, thus leading to lysosomal damage and the leakage of cathepsins into the cytoplasm and, consequently, to NLRP3 inflammasome activation [68].

Studies have demonstrated that the expression levels of NLRP3 inflammasome components are upregulated in atherosclerosis, which are potential links to the severity of the disease [68]. Furthermore, studies using bone marrow-transplanted mice, lentivirus-mediated NLRP3 gene silencing or the selective NLRP3 inhibitor MCC950 in apoE^−/−^ mice have provided direct evidence that the NLRP3 inflammasome contributes to the progression of atherosclerosis [69]. However, there are conflicting data showing no significant differences in plaque size or macrophage infiltration between apoE^−/−^ mice and apoE^−/−^ mice deficient in NLRP3 or caspase-1 [70]. Very recently, Chen et al. reported that a lack of NLRP3 in bone marrow cells in LDL receptor^−/−^ mice attenuated atherosclerotic lesion formation in female mice but not in male mice [71]. Further research is needed to explore the potential role of the NLRP3 inflammasome in the development of atherosclerosis, also considering sex differences.

### 4.2. Neutrophils’ Healing Potentiality

Despite this evidence, almost all therapeutic strategies aiming to contrast neutrophils have thus far failed to show clinical benefits [72]. Experimental studies have reported that long-term depletion of neutrophils in MI models did not improve but rather impaired the inflammation process, leading to negative remodeling and reduced cardiac function [73].

The emerging role of neutrophils in the reparative process and healing after an injury has been reported in the last few years. Neutrophils contribute to the biosynthesis of growth factors, including VEGF, and factors favoring the inflammation switch off, such as lipoxins, resolvins, and protectins [74,75]. Therefore, general anti-neutrophil approaches can limit acute post-ischemic tissue injury, but this potential benefit is counterbalanced by the blockage of the subsequent healing response. In a murine model, Horckmans et al found that neutrophil-depleted mice subjected to MI had worsened cardiac function, increased fibrosis, and progressively developed heart failure, enlightening the crucial role of neutrophils into macrophage polarization as a healing phenotype [76]. Similarly, long-term inhibition of the S100 A8/A9 heterodimer has led to the deterioration of cardiac function over time [77].

The promising results observed in the CANTOS trial [78] and COLCOT trial [79] should drive future intervention and research in inflammatory regulation among ACS patients (Table 1). These two large trials inquired about the effect of canakinumab and colchicine in patients with recent ACS, finding benefits in terms of reductions in major cardiovascular adverse events, mainly driven by reductions in ischemic events. The inhibition of the IL-1β pathway has shown positive prognostic impacts after MI, even if some contrasting results have been reported by other drugs used to target the same pathways, such as in the MRC-ILA Heart Study, which failed to demonstrate a benefit of treatment with anakinra, a IL-1 receptor inhibitor, in NSTEMI patients [80]. Advances in understanding neutrophil biology imply a tailored approach to target not only the mechanisms and actors involved, but also the time and duration of the treatment, in order to act without damage [81].

### 4.3. Adaptive Immunity

Alongside the role of innate immunity, T and B lymphocytes operate in an elaborate web of signaling and subtype differentiation in the atherosclerosis context [94]. The adaptive immune response is mediated toward lipoprotein components trapped in the artery wall and toward proteins coming from dysfunctional endothelial cells [95].

In the early stages of atherosclerosis, the recruitment of CD4+ T cells with a regulatory phenotype (Treg) rather than an effector phenotype (Teff) is predominant [96]. In an animal model of hypercholesterolemia, a rise in Treg in atherosclerotic plaque has been observed with a concomitant lower number of Teff [97]; moreover, a depletion of CD4+ T cells may accelerate atherosclerosis in mice [98], promoting the concept of an initial atheroprotective role played by CD4+ T cells. However, the determinants of the switch of T lymphocytes from a protective role to atherogenic action are not completely defined. A combination of increased inflammatory cytokines, recruitment chemokines, reduced coinhibitory molecules and anti-inflammatory mediators may be responsible for the variation in the T cell subset proportions in the atherosclerosis milieu [99]. CD4+ T cell subpopulations can differentially impact atherosclerosis progression and ACS events [100]. Recent evidence has suggested T cell plasticity in addition to their heterogeneity. A specific CD4+ T cell subtype can acquire the transcriptomic and phenotypic properties of another subtype [96,101]. A persistent atherogenic condition can influence Treg by modulating the forkhead box P3 (FoxP3) gene, essential for anti-atherogenic actions. Treg might progressively loose the FoxP3 expression in favor of an effector and pro-atherothrombotic phenotype [102].

The role of B lymphocytes is less well characterized, but their contribution in atherosclerosis has been depicted [103]. A primary protective role has been hypothesized, even if the recent definition of B1 and B2 subtypes has provided controversies regarding their role. In particular, the B1 subtype was confirmed to be anti-atherogenic by producing immunoglobulin M antibodies to recognize apoptotic cells and to oxidize LDL [104]. The contribution of B2 lymphocytes still needs more clarification. Their antibody production is mainly derived from germinative centers, and has displayed the capacity to promote atherosclerosis [105]. B2 cells-derived immunoglobulin G antibodies with pathogenic role have been described as recognizing heat shock proteins 60 and 65, released from a damaged endothelium and enhancing inflammation [95].

Increased attention is warranted regarding perivascular adipose tissue (PVAT) as an active paracrine modulator of inflammation and adaptive immunity. A number of different lymphocyte subtypes have been found in the adipose tissue located on the external surface of arteries, including the coronaries [106,107]. The interplay between PVAT and atherosclerosis is mediated by adiponectin, adipokines and other cytokines [108]. Initially, PVAT exerts a protective action against endothelial dysfunction and conditions promoting atherosclerosis by the production of vasoactive immunoregulatory substances [109]. Impaired PVAT homeostasis, such as in obesity or systemic inflammation, leads to the loss of brown adipocyte components, with consequent atherosclerotic plaque progression [110]: the following infiltration of immune cells, including CD4+ T cells with effector polarization aggravates lesion severity and the stability of plaque [111,112].

## 5. Metabolic and Lipid-Related Actors in the Atherothrombosis Process

### 5.1. Adenosine Pathways

The first evidence of adenosine involvement in the cardiovascular system was reported by Berne [113], who described its contribution in coronary vasodilation. Alongside the electrophysiological properties in the diagnosis and treatment of wide QRS tachycardia and supraventricular tachycardia, adenosine has been found to be involved in several pathways related to coronary blood flow and atherothrombotic processes. Effects of adenosine are mediated by four different G protein-coupled receptors, but the major cardiovascular role is played by the A2a type, which is widely expressed in smooth muscle and the endothelial cells of vessels. The protective role in atherosclerosis is constituted by the inhibition of the pro-inflammatory cytokine response [114], together with the stimulation of endothelial cell proliferation during angiogenesis [115]. The promotion of collateral circulation is crucial against ischemia-induced damage. Hypoxia has been reported to increase A2a receptor levels [116]. Upregulation of the A2a receptor counterbalances the inflammatory response mediated by NF-kB and hypoxia-induced factor (HIF)-1α pathways [117].

Another key role of adenosine and the A2a receptor is the regulation of platelet aggregation. In an animal model, mice knockout for the A2a receptor displayed higher platelet aggregation: the effect is mediated by an increase in platelet intracellular cAMP concentration with a consequent reduction in internal calcium mobilization [118]. The magnitude of adenosine impact on platelet aggregation is not negligible: for example, the direct P2Y_12_ antagonist ticagrelor inhibits equilibrate nucleoside transport 1, with a consequent increase in plasmatic adenosine levels [119]; moreover, genetic polymorphism of the A2a receptor has shown significantly different platelet reactivities among patients on ticagrelor [120]. Similarly, the procedural use of adenosine during the assessment of FFR deserves careful attention in relation to dosage of intracoronary adenosine [121] and genetic variants that may affect the final results [122]. 

### 5.2. Lp(a)

Lp(a), an apolipoprotein B (apoB)-containing lipoprotein similar to LDL, is bound to apolipoprotein(a) (apo(a)) on its surface. Apo(a) is a protein encoded by the lipoprotein(a) gene and possesses significant homology with plasminogen [123]. Lp(a) has various physiological and pathological roles, including modulation of coagulation, immune cell interaction, and proliferation of vascular smooth muscle and adhesion molecules. It is also a major carrier of oxidized phospholipids in human plasma, which can have pro-atherogenic and pro-inflammatory effects [123].

In terms of disease, Lp(a) contributes to the formation and progression of atherosclerotic plaques, potentiates thrombus formation to cause MI or ischemic stroke, and promotes inflammation and calcification [124].

The population distribution of circulating Lp(a) concentration is positively skewed, strongly supporting a relationship between raised Lp(a) and the risk of CAD and acute events [125]. Mendelian randomization studies indicate a causal relationship between the lifelong elevation of Lp(a) and MI, with the doubling of Lp(a) plasma levels resulting in a 22% increased risk of MI [126].

The cholesteryl ester transfer protein (CETP) inhibitors anacetrapib and evacetrapib reduced Lp(a) by 34 % and 40%, respectively, in two different phase II trials [82,84]. These drugs did not demonstrate an overall benefit for CVD risk reduction but, importantly, were evaluated in trials that did not enroll patients on the basis of elevated Lp(a). Even when a large study, the REVEAL trial, found a lower incidence of major coronary events in patients treated with anacetrapib than with the use of placebo [83], the overall benefits and cost–benefit ratio hampered to proceed for the introduction in daily practice [127].

Lipoprotein apheresis offers an effective means of lowering apoB-containing lipoproteins, including LDL and Lp(a). It can reduce both Lp(a) and LDL-C from baseline by about 70% even if the reduction in Lp(a) is inconstant and time-sensitive [85].

### 5.3. Vitamin D and Calcium Homeostasis

Vitamin D is a lipophilic vitamin primarily involved in the homeostasis of calcium, phosphorus and bone tissue, but it also has a wide range of systemic hormonal effects and organ targets, including the cardiovascular system [128]. Cholecalciferol is the compound named vitamin D3, and it is synthesized in the skin upon ultraviolet waves or is derived from food as ergocalciferol. It undergoes first modification in liver cells, receiving a hydroxyl group on C25 and becoming 25(OH) vitamin D; the active form requires further hydroxylation on C1, occurring mainly in the kidneys and other extrarenal sites, with the formation of 1,25(OH)2 vitamin D, named calcitriol [129]. The vitamin D receptor binds vitamin D in the form of 1,25(OH)2 and is expressed widely in the cardiovascular system [130], promoting the signaling cascade and its effects. The cardiovascular protective role of vitamin D has been shown in several studies, which have all elucidated that vitamin D counteracts inflammation and oxidative stress and is a cornerstone of atherosclerosis [131,132,133].

Moreover, among ACS patients, calcitriol deficiency was found in about 9% of patients, even among those with normal 25(OH) vitamin D. A significant inverse relationship of calcitriol with inflammatory and metabolic biomarkers has been reported, suggesting a potential accurate role of calcitriol to predict cardiovascular risk [134]. Another interesting finding is the inverse relationship between vitamin D level and pro-atherogenic lipid profile and the burden of lesion calcification in patients admitted for STEMI [135].

This evidence is in line with and supports the concept of the bone–vascular axis [136,137,138]. Prior studies have found that the increase in bone resorption occurring in elderly and post-menopausal women leads to calcium mobilization and subsequent abnormal deposition in vessels walls [139]. Several mediators including fibroblast growth factor, osteopontin, osteoprotegerin, and matrix proteins have shown a connection between bone homeostasis and vascular calcification [140]. Subsequent modification in vessel components affects smooth muscle cells. When they are exposed to extracellular vesicles from bone with impaired instead of normal metabolism, vascular smooth muscle cells might undergo osteogenic transition with consequent vessel calcification [141]. This so-called calcification paradox was also found in settings different from primary bone affection, such as osteoporosis. In chronic kidney disease, osteogenic transition has been observed [139,142], claiming a role in increased serum phosphorus levels or decreased hydroxylation of 25(OH) vitamin D. Whether calcium deposition in coronary vessels should be considered a risk factor or protective for acute events is still under discussion [143]. These crucial advances in preclinical studies deserve a transition in the clinical setting.

Clinical trials have explored the cardiovascular effects of vitamin D supplementation, reporting contrasting results. Administration of cholecalciferol compared to placebo in patients with hypovitaminosis D has shown no changes in various cholesterol and lipoproteins levels [86]. Moreover, two large trials, ViDA [87] and VITAL [88], enrolled 5110 and 25,871 patients, respectively, to assess the effect of the supplementation of cholecalciferol in healthy adults. No benefit was found in terms of the reduction of major cardiovascular adverse events, including cardiovascular death, over a follow-up period of 3.3 and 5.3 years. Meta-analyses of randomized trials in CAD patients have confirmed these inconclusive results, portraying the need for further investigation [88].

### 5.4. Lipoprotein-Associated Phospholipase A2

The term phospholipase A2 (PLA2) refers to a superfamily of phospholipase enzymes that hydrolyze the ester bond at the sn-2 position of phospholipids. After hydrolyzation, PLA2 releases free fatty acids. A proportion of them includes arachidonic acid and oleic acid, crucial compounds for energy production and inflammation signaling pathways [144].

The PLA2 superfamily comprises 16 groups identified according to the differences in the chronology of their discovery [145]. Group VII includes lipoprotein-associated PLA2 (Lp-PLA2). It is secreted by macrophages, monocytes, mast cells and T-lymphocytes, and it circulates in the blood in the form of a complex with LDL and high-density lipoprotein [146]. Lp-PLA2 was originally called platelet-activating factor acetyl-hydrolase, because of its hydrolytic action on platelet-activating factor [147].

These effects produce an acceleration of atherosclerosis and plaque development, including the formation of a necrotic core. Increased levels of Lp-PLA2 are found in thin-cap.

Several studies have reported that high plasma Lp-PLA2 levels are independently associated with an increased risk of CAD and stroke [148,149,150]. Moreover, in a murine model, the downregulation of the group VII of PLA2 genes improves inflammation and reduces the burden of atherosclerosis [151], suggesting Lp-PLA2 as a potential target for therapeutics. However, despite the pro-atherogenic effect and prediction of adverse events, the inhibition of Lp-PLA2 with darapladib, the most advanced and most widely studied Lp-PLA2 inhibitor, has failed to provide substantial benefits in patients with stable CAD [152] and admitted for an ACS [89]. In particular, in the SOLID-TIMI 52 trial, more than 13,000 subjects were randomized to receive either once-daily darapladib or placebo within 30 days of hospitalization for an ACS. With a median follow-up of 2.5 years, the primary endpoint, major coronary events, occurred in 16.3% of patients in the darapladib group and in 15.6% in the placebo group (HR = 1.00, 95% CI: 0.91–1.09, *p* = 0.93) [89].

Some explanations have been proposed to understand the discrepancy between pre-clinical evidence and the lack of clinical benefits. Patients enrolled in the darapladib clinical trials displayed higher percentages of statin therapy, which may reduce the Lp-PLA2 level and limit the magnitude of further Lp-PLA2 reduction through specific inhibitors. Concerns from other authors have involved the absence of stratification of patients according to residual inflammation after an acute event that leads to hospitalization, suggesting the need for a more accurate selection of patients potentially benefiting Lp-PLA2 inhibition [153]. Therefore, Lp-PLA2 may be considered as a reliable biomarker of cardiovascular risk, rather than a causal element of atherosclerosis [154].

## 6. Genetics: The Different RNAs in ACS

### 6.1. MicroRNA

MicroRNAs (miRNAs) play an essential role in cardiovascular biology, with dysregulation linked to various pathologies [155]. Several miRNAs have been found to be altered in cardiovascular diseases [156], and cardiac-specific miRNAs have been reported to contribute to the development of CAD, with evidence of differences in miRNA expression between CCS and ACS [157] (Table 2).

From a pathological point of view, miRNAs have been found to be related to alterations involving several key actors of atherosclerosis. A number of miRNAs, including miR-34a, miR-217, and miR-146a, have been associated with regulating mechanisms involved in endothelium senescence [189]. A decrease in sirtulin-1, a gene of longevity, was identified as a main effect of miRNA interaction [190]. On the other hand, some mrRNAs have shown the ability to control inflammation and reduce leukocyte infiltration in the vessel wall and plaques, such as miR-126 [191]. In addition, the homeostasis of smooth muscle has been found to be influenced by miRNAs. miR-143 and miR-145 may promote the differentiation of smooth muscle cells and have been found to be reduced in atherosclerotic vessels [192].

A number of cardiac- and muscle-specific miRNAs were proposed as biomarkers of acute MI in pivotal studies [193,194]. In particular, mir-208b and mir-499 were found to be significantly effective in predicting MI compared to controls, with an AUC = 0.97 for mir-499, even if no adjunctive impact to troponin diagnostic accuracy was shown [165]. Subsequent studies have confirmed the role of mir-499 in the prediction of acute MI together with mir-133a [169], while mir-208b was suggested to be further investigated [195]. In a study including more than three hundred ACS-suspected patients, cardiac and non-cardiac miRNAs were assessed. The levels of all miRNAs studied were significantly increased in 106 patients diagnosed with ACS, even among those with first high-sensitive troponin that resulted in negative or early symptom onset (i.e., less than 3 h). Cardiac miR-1, miR-499 and non-cardiac miR-21 were found to increase the diagnostic value when added to troponin, independent from patient comorbidities and cardiovascular risk factors. Moreover, the combination of the three miRNAs resulted in a significantly higher AUC than troponin among ACS patients earlier admitted or referred to hospital [159].

In addition, inflammation-related miRNAs have been inquired for their potential relationship with ACS, including miR-181c and miR-362 [196]. The levels of these miRNAs were found to be higher in ACS patients, while other authors reported no increased levels among stable CAD [197]. However, their expression was not found to be related to troponin levels, suggesting a role of these inflammatory miRNAs to be more on plaque vulnerability than on the extent of myocardial injury.

The wide and still unexplored field of miRNAs may be hiding some concerns, and findings must be interpreted with caution. There are several potential biases related to the type of body fluid used for miRNA extraction, sample preparation, platform for analysis, RNA purification, and normalization methods, of which all can affect the concentration and quality of miRNAs [198]. In addition, miRNAs selection that has adopted candidate-driven approaches might lead to biases regarding the magnitude of the effect estimation [199]. Therefore, it is essential to interpret the findings of miRNA studies with caution, as there is an absence of standardization when it comes to selecting miRNAs, normalization methods, and RNA extraction techniques [200].

### 6.2. Long Non-Coding RNAs

Long non-coding RNAs (lncRNAs) have been shown to play a role in a variety of cellular functions within both the nucleus and cytoplasm and are important for normal development as well as in the progression of disease [201]. RNA-seq studies have been performed to assess lncRNA expression, both in animal models and human tissues [202,203], in the context of cardiovascular disease [204]. For example, a study using RNA-seq to investigate the dynamic regulation of lncRNAs in ischemic heart disease in pigs identified 450 lncRNAs that were dysregulated in cardiac tissue after MI [205].

Additionally, two novel, hypoxia-sensitive human endothelial lncRNAs were discovered using RNA-seq and microarray technologies, which upon further functional characterization through gain- and loss-of-function approaches, were found to have an important role in angiogenesis [206]. Furthermore, the potential value of lncRNAs as diagnostic biomarkers has been explored, and a global transcriptomic analysis of plasma samples from patients with MI led to the identification of 768 lncRNA transcripts that were dysregulated compared to control samples [207]. In particular, the mitochondrial lncRNA uc022bqs.1 (LIPCAR) was found to be downregulated immediately after MI but upregulated during later stages of cardiac damage, suggesting that it could be used to identify patients who are prone to cardiac remodeling following a MI [207].

In a case-control study, HIF-1α antisense RNA 2 (aHIF), KCNQ1 overlapping transcript 1 (KCNQ1OT1), and metastasis-associated lung adenocarcinoma transcript 1 were significantly increased in circulation in patients with acute MI, with further differences in circulating levels according to STEMI or NSTEMI presentation [181].

LncRNA myosin heavy-chain-associated RNA transcripts (MHRT) and other less common lncRNA were proposed as a potential diagnostic tool in the setting of acute MI [208]. Zhang et al., in their study, found increased levels of MHRT among patients referred for ACS compared to healthy volunteers. Moreover, they explored in murine MI models the role of lncRNA MHRT, reporting a protective role against hydrogen-peroxide-induced myocytes apoptosis [182].

Of interest is another lncRNA named urothelial carcinoma-associated 1 (UCA1). It was evaluated by Yan and colleagues in a case-control investigation comparing acute MI patients and healthy control [183]. Their main findings displayed a significant reduction in plasmatic UCA1 levels in the early phase of MI but a significant increase at day 3 after MI, irrespective of hypertension or diabetes diagnosis. UCA1 has been found to play a role in glucose metabolism [209], cell proliferation and inhibition of apoptosis [210], and most cardiac protective actions derive from their interaction with miR-1. Additive value to troponin and other myocardial injury biomarkers has been proposed for UCA1 [183].

### 6.3. Circular RNAs

Circular RNAs (circRNAs) are a significant class of non-coding RNA molecules that form a covalently closed loop. Spliceosome machinery is responsible for the production of circRNAs through the process of precursor mRNA back-splicing [211]. High-throughput sequencing has identified thousands of circRNAs present in the human body and has demonstrated that a substantial number of these are expressed in a tissue-specific or disease-specific manner [212,213,214]. While the biological functions of the majority of circRNAs are unknown, certain circRNAs have been reported to serve as miRNA sponges [215,216], to interact with RNA-binding proteins, to regulate transcription or to translate into proteins [217,218]. Early research has provided evidence of individual circRNAs playing pivotal regulatory roles in the cardiovascular system [219,220].

Key mechanisms of impact of circRNAs on MI involve oxidation-stress-induced cardiomyocyte apoptosis [221,222], with co-participation of miRNAs such as miR-133a [223], and ischemia-reperfusion-induced damage and cell death. The circRNA MFACR directly promotes mitochondrial fission and ischemia reperfusion apoptosis by interacting with miR-652.

Murine models of MI have been used by Geng et al. to explore the role of circRNA Cdr1as, also named CiRS-7, in infarcted cardiomyocytes. Their main findings were consistent with an increased expression of Cdr1as and cardiac infarct size and cell apoptosis promotion. Its effect was counterbalanced by miR-7a expression, which in turn decreased cell apoptosis and reduced the infarct size [184]. In in vitro models of acute MI, the circRNA circ-tetratricopeptide repeat domain 3 (circ-Ttc3) was found to be significantly upregulated, contributing to the reduction of ATP depletion and cell death. Proposed mechanisms include a potential sponge role of circ-Ttc3 with miR15b-5p, which in turn increases the expression of ADP ribosylation factor-like GTPase 2 (Arl2) protein, the main effector with a beneficial role shown by circ-Ttc3 [186]. A protective role against autophagy has been shown by autophagy-related circular RNA (ACR) [188], while an intriguing role in supporting myocardium healing after MI has been proposed with the co-participation of VEGF and connective tissue growth factor [224,225], as well as its role in the regulation of angiogenesis [185].

Despite the promising findings from pre-clinical studies, the still poor understanding of the biology of circRNA hampers its complete translation into the clinical field, requiring clarification both in their synthesis and activities [226].

## 7. Example of Genic Therapy

### 7.1. Olpasiran

Olpasiran is a siRNA drug administered subcutaneously and targeting the liver via its N-acetylgalactosamine moiety. Upon entering the hepatocyte, the antisense strand of olpasiran is loaded into an RNA-induced silencing complex (RISC), which binds to apolipoprotein(a) RNA messenger (mRNA), leading to its degradation. This allows for a prolonged effect, as the RISC can target additional mRNAs [227]. Clinical trials have shown that olpasiran treatment significantly reduces the concentration of Lp(a) in a dose-dependent manner and is safe, with doses reducing the Lp(a) concentration by more than 95% compared with placebo [228]. The pharmacodynamic effects were maintained when the drug was administered every 12 weeks. Although there is a causal role for Lp(a) in atherosclerotic cardiovascular disease [229], large-scale clinical trials are required to demonstrate a clinical benefit of Lp(a) lowering [230,231]. The relationship between Lp(a) and the risk of atherosclerotic cardiovascular disease appears to be broadly continuous and log-linear, making it difficult to identify a clear threshold for patient risk [232].

### 7.2. Inclisiran

For the treatment of heterozygous familial hypercholesterolemia or CAD requiring additional LDL lowering, inclisiran can be used as an adjunct to diet and maximally tolerated statin therapy [233]. This siRNA inhibitor of proprotein convertase subtilisin/kexin type 9 synthesis is administered via subcutaneous injection every three months in two doses, then every six months thereafter.

Results from the ORION-9, -10, and -11 phase III trials, published in 2020, have demonstrated a placebo-corrected percentage change in LDL from baseline to day 510 of 47.9 percent (95% CI 42.3–53.5) and a time-adjusted reduction of 44.3 percent (95% CI 40.1–48.5) [90,91,92,234]. As such, this may be a viable alternative for those with allergic responses to both evolocumab and alirocumab, as well as for those with difficulty using a pen injector due to arthritis and/or weakness of the hands. However, further clinical trial data are required in order to assess the potential for cardiovascular risk reduction before a broader use of inclisiran is suggested.

The ORION-10 and ORION-11 trials showed that inclisiran significantly reduced LDL levels by approximately 50 percent in patients with cardiovascular disease and at high risk. The pooled analysis results of these trials indicate that the placebo-corrected change in LDL with inclisiran at day 510 was −50.7 percent, with few serious adverse events reported [235].

## 8. Future Perspectives

**Figure 3:**
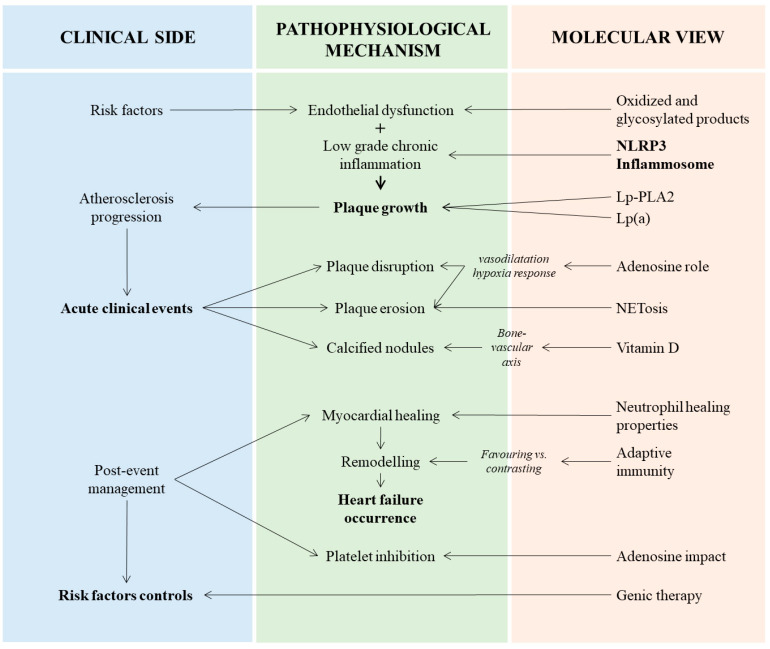
Interplay between clinical manifestations, pathophysiological mechanisms and main actors related to atherosclerosis in acute coronary syndromes. In the left column are the three main clinical steps of acute coronary syndrome: pre-clinical atherosclerosis progression, acute event occurrence and post-event management. In the center column are the principal signaling pathways and underlying pathological aspects. In the right column are the key elements and substances mediating the pathophysiological process. Lp(a), lipoprotein(a); Lp-PLA2, lipoprotein associated-phospholipase A2; NETosis, neutrophil extracellular traps (NETs) formation; NLRP3, nucleotide-binding oligomerization domain-like receptor family pyrin domain containing 3.

The significant advances achieved in recent years have contributed to a finer comprehension of atherosclerosis pathophysiology (Figure 3). The paradigm of ruptured culprit lesions as the only cause of ACS has shifted to a more complex series of mechanisms that probably deserve different approaches from the interventional and non-interventional points of view. On the side of pharmacological strategies, the characterization of the neutrophils’ role could provide attractive targets of treatment, even if a further definition on the best way and time to treat is required to preserve their potential healing properties. Clinical translation of pre-clinical findings in metabolic- and lipid-related factors should be considered and performed, with adequate identification of the subgroups of patients that might take advantage from the treatment. Finally the great advances in genetics have provided a large field of investigation. The amount of data is impressive, but a better definition is needed to understand the potential benefits of all non-coding RNAs that have been investigated.

## Figures and Tables

**Table 1:** Main clinical study and evidence in humans of the discussed targets.

Target	Drug/Approach	Study Name and Type	Comparator(s)	Population	Evidence
Lp(a)	Anacetrapib	0859-026 AM3—phase II RCT	Atorvastatin, atorvastatin + placebo, placebo	39 mildly hypercholesterolemic patients	Reduction of Lp(a) by 34.1% [82]
		REVEAL—phase III RCT	Placebo	30,449 adults who were receiving intensive atorvastatin therapy	Lower incidence of major coronary events vs placebo [83]
	Evacetrapib	Nicholls et al.—phase II RCT	Placebo	393 mildly hypercholesterolemic patients	Reduction of Lp(a) by up to 40% [84]
	Apheresis	Leebmann et al.—observational study		170 high risk patients	Potential benefit in lower incidence of cardiovascular events [85]
	Oplisiran	OCEAN(a)-DOSE trial—phase II RCT	Placebo	281 patients with established atherosclerotic	Reduction of Lp(a) concentration
Vitamin D pathways	Cholecalciferol	Ponda et al.—RCT	Placebo	151 vitamin D deficient adults	No improvement of the lipid profile [86]
		ViDA—RCT	Placebo	5110 healthy subjects	No difference in major cardiovascular event rate [87]
		VITAL—RCT	Factorial 2 × 2, omega-3 vs. placebo, x cholecalciferol vs. placebo	25,871 healthy subjects	No difference in major cardiovascular event rate [88]
Lp-PLA2	Darapladib	SOLID-TIMI 52—RCT	Placebo	13,000 subjects with recent ACS	No differences in major cardiovascular events [89]
PCSK9	Inclisiran	ORION-1—phase II RCT	Placebo	501 patients at high risk for cardiovascular disease	Reduction of LDL cholesterol [90]
		ORION-9—phase III RCT	Placebo	482 patients with heterozygous familial hypercholesterolemia	Reduction of LDL cholesterol [91]
		ORION-10—phase III RCT	Placebo	1561 patients with atherosclerotic cardiovascular disease	Reductions in LDL cholesterol levels of 50% [92]
		ORION-11—phase III RCT	Placebo	1617 patients with atherosclerotic cardiovascular disease or risk equivalent	Reductions in LDL cholesterol levels of 50% [92]
Il-1β	Canakinumab	CANTOS—RCT	Placebo	10,061 patients with prior MI	Reduction of major cardiovascular events [78]
IL-1 and inflammasone	Colchicine	COLCOT—RCT	Placebo	4745 patients with MI	Reduction of first and total ischemic cardiovascular event [79]
		LoDoCo2—RCT	Placebo	5522 patients with CCS	Reduction of cardiovascular event [93]
IL-1 receptor	Anakinra	MRC-IL1-HEART—phase II RCT	Placebo	182 individuals with early NSTEMI	No difference in major cardiovascular event rate; warning for harms at long follow-up [80]

ACS, acute coronary syndrome; CCS, chronic coronary syndrome; IL, interleukin; LDL, low-density lipoprotein; Lp(a), lipoprotein(a); Lp-PLA2, lipoprotein associated-phospholipase A2; MI, myocardial infarction; NSTEMI, non-ST segment elevation myocardial infarction; PCSK9, proprotein convertase subtilisin–kexin type 9; RCT, randomized controlled trial.

**Table 2:** Main non-coding RNAs and cardiovascular conditions related to acute coronary syndrome.

Pathological Condition	*microRNAs*	Action/Regulation
Ischemia reperfusion injury	miR-146a	Regulation of IRAK1 and TRAF6 expression in the myocardium [158] increased expression in acute MI patients [159]
	miR-17-3p	Reduction of PTEN signaling [160]
Atherosclerosis	miR-100	Regulation of mTOR signaling, promoting endothelial autophagy and reducing NF-kB activation [161]
	miR-181b	Regulation of TIMP3 [162]
	miR-181a and miR-181c	increased expression in acute MI patients [163,164]
Myocardial infarction	miR-208b	Increase expression in acute MI patients and lower in stable angina pectoris [165,166]
	miR-99a	Regulation of mTOR–p70S6K signaling pathway [167]
	miR-199a-3p	Promotion cardiac regeneration [168]
	miR-590-3p	Promotion cardiac regeneration [168]
	miR-499	Increased expression in acute MI [165,169]
	miR-133a	Increased expression in acute MI [169]
	miR-31	Regulation of troponin, E2F6 and mineral corticoid receptor [170]
Cardiac fibrosis	miR-433	Regulation of ERK-p38 kinase pathway, involved in MAPK8 signaling [171]
	miR-378	Regulation of MKK6 [172,173]
	miR-21	Regulation ERK-MAPK pathway [174] Increased expression in ACS [159]
**Pathological** **Condition**	***Long non-coding RNAs***	**Action/Regulation**
Myocardial infarction	MIRT1	Downregulation NF-kB signaling [175]
	UIHTC	Regulation of mitochondrial function [176]; protection of myocardial cells from apoptosis [176]
	AZIN2-sv	Inhibition PI3K-AKT(PKB) pathway [177]
	MEG3	Promotion hypoxia-induced apoptosis [178]
	HOTAIR	Promotion hypoxia-induced apoptosis [179,180]
	KCNQ1OT1	Regulation K channel expression [181]
	aHIF	Regulation HIF-1α [181]
	MHRT	Protection against H2O2–induced apoptosis [182]
	UCA1	Metabolic regulation and cell proliferation [183]
**Pathological** **Condition**	***Circular RNAs***	**Action/Regulation**
Myocardial infarction	Cdr1as (CiRS-7)	Regulation of miR-7a pathways Increase in the infarct size in murine model [184]
	CircNfix (mmu-circ-0001704)	Inhibition of cardiomyocyte proliferation, promotion of cardiac dysfunction [185]
	Circ-Ttc3	Inhibition of ATP depletion and cell death in acute MI, through miR15b-5p-ARl2 pathways [186]
Ischemia reperfusion injury	MFACR (mm9-circ-016597)	Downregulation of miR-652-3p, which in turn suppresses MTP18 translation; promotion of mitochondrial fission and apoptosis [187]
	ACR (mmu-circ-006636)	Suppression autophagy [188]

ACS, acute coronary syndrome; ACR, autophagy-related circular; aHIF, HIF-1α antisense RNA 2; AZIN2-sv, antizyme inhibitor 2 splice variant; ERK, extracellular signal-regulated kinase; HIF-1α, hypoxia induced factor-1α; HOTAIR, transcription in trans across 40 kb of the HOXD locus; IRAK1, IL-1 receptor-associated kinase 1; KCNQ1OT1, KCNQ1 overlapping transcript 1; MAPK, mitogen-activated protein kinase; MEG3, maternally expressed gene 3; MFACR, mitochondrial fission and apoptosis-related circRNA; MHRT, myosin heavy-chain-associated RNA transcripts; MI, myocardial infarction; MIRT1, myocardial infarction-associated transcript 1; MKK6, mitogen-activated protein kinase kinase 6; mTOR, mechanistic target of rapamycin; MTP18, mitochondrial fission process protein 1; NF-κB, nuclear factor-κB; PI3K, phosphatidylinositol 3-kinase; PKB, protein kinase B; PTEN, phosphatase and tensin homologue; TIMP, metalloproteinase inhibitor; TRAF6, TNF receptor-associated factor 6; UCA1, urothelial carcinoma-associated 1; UIHTC, upregulated in hypothermia-treated cardiomyocytes.
